# A 6-DOF Navigation Method based on Iterative Closest Imaging Point Algorithm

**DOI:** 10.1038/s41598-017-17768-2

**Published:** 2017-12-12

**Authors:** Shuai Shi, Zheng You, Kaichun Zhao, Zhaoyao Wang, Chenguang Ouyang, Yongkui Cao

**Affiliations:** 1 Department of Precision Instrument, Tsinghua University, Beijing, China; 2 State Key Laboratory of Precision Measurement Technology and Instruments, Tsinghua University, Beijing, China; 3 China Manned Space Agency, Beijing, China

## Abstract

To achieve six degree-of-freedom autonomous navigation of an inboard spacecraft, a novel algorithm called iterative closest imaging point (ICIP) is proposed, which deals with the pose estimation problem of a vision navigation system (VNS). This paper introduces the basics of the ICIP algorithm, including mathematical model, algorithm architecture, and convergence theory. On this basis, a navigation method is proposed. This method realizes its initialization using a Gaussian mixture model-based Kalman filter, which simultaneously solves the 3D-to-2D point correspondences and the camera pose. The initial value sensitivity, computational efficiency, robustness, and accuracy of the proposed navigation method are discussed based on simulation results. A navigation experiment verifies that the proposed method works effectively. The three-axis Euler angle accuracy is within 0.19° (1σ), and the three-axis position accuracy is within 1.87 mm (1σ). The ICIP algorithm estimates the full-state pose by merely finding the closest point couples respectively form the images obtained by the VNS and predicted at an initial value. Then the optimized solution of the imaging model is iteratively calculated and the full-state pose is obtained. Benefiting from the absence of a requirement for feature matching, the proposed navigation method offers advantages of low computational complexity, favorable stability, and applicability in an extremely simple environment in comparison with conventional methods.

## Introduction

**Figure 1:**
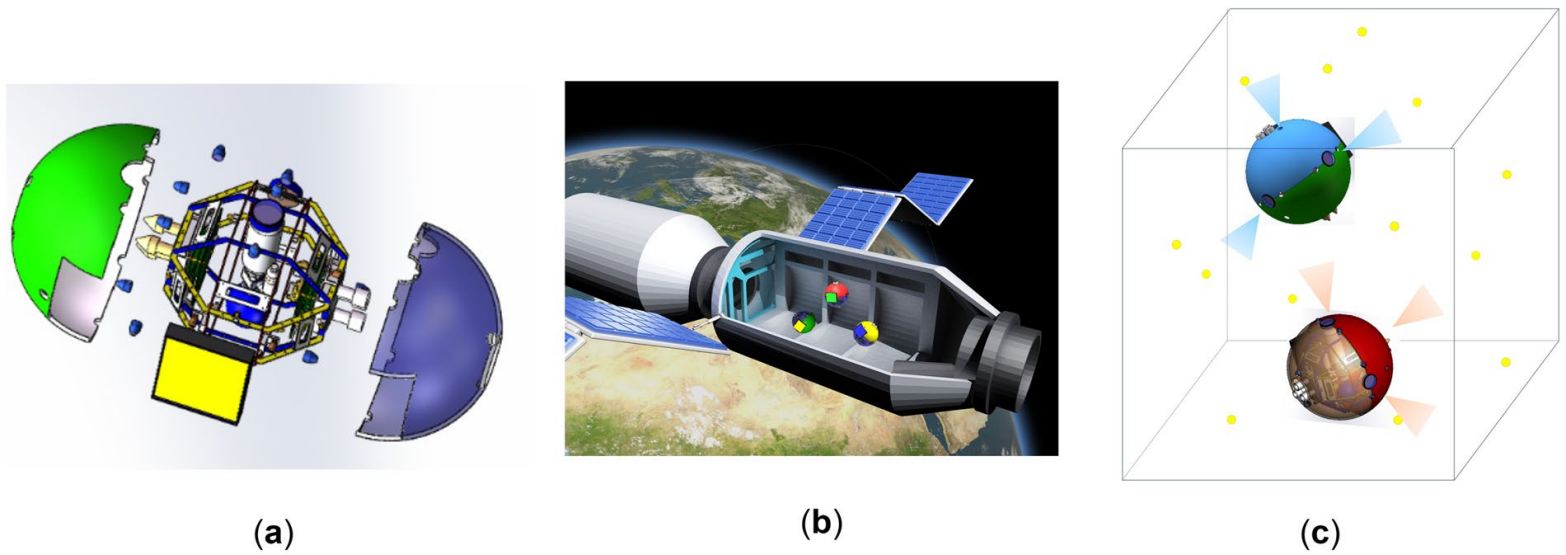
(**a**) 3D model, (**b**) flying sketch and (**c**) navigation sketch of the IS.

The inboard spacecraft (IS) of Chinese Tiangong space station (Fig. 1) was proposed by Tsinghua University and China Manned Space Agency in 2015 to conduct algorithm evaluation and on-orbit service demonstration^1^. As the requirements of high-precision navigation and detection capability are considered, computer vision is an excellent choice for IS compared with other available navigation methods, such as infrared^2^, ultrasound^3^, UWB^4^, and Wi-Fi^5^. Navigation is one of the most important applications of computer vision and has been the focus of research since its first proposal. The existing vision navigation methods can be simply classified into two categories according to the known/unknown environment where the user is situated. Research on navigating in known environments is discussed by Rudol P^6^, tawdross P^7^ and Serrão M^8^, whereby the features of the known environment are previously obtained and used as navigation references. Other researchers have attempted to solve navigation problems in an unknown environment. Methods based on optic flow^9,10^ and SLAM^11,12^ are explored in depth.

On the basis of the IS mission plan, navigation inside the space station aims to achieve the following:

1. use of the cabin frame, which is attached to the space station, as the reference frame;
2. 6-degree of freedom (DOF) full-state parameter estimation throughout the flight of the IS; and
3. low computational complexity.

The IS presents 6-DOF free flight inside the space station, where the surrounding area is known and stable (Fig. 1(b)). Thus, the features attached inside the cabin can be used as navigation references. The coordinates of the features in the cabin frame are essential to achieve the aforementioned requirement 1). Point features are good choices for navigation references because they are easy to deploy inside the cabin and their 3D coordinates are convenient to measure (Fig. 1(c)). These points, which are vision navigation beacons, can be artificially designed to become easily distinguishable from the environment through features such as shape, gray scale and polarization degree. Therefore, their images can be efficiently extracted from the pictures. The database of the beacon coordinates and features can also be uploaded to the IS before its flight and learning the cabin environment is not necessary, thereby meeting requirement 3). To address requirement 2), a sufficient number of beacons should be deployed reasonably.

Unfortunately, although the cooperative beacon points meet the basic requirements, challenges to realize IS navigation remain with existing methods. The number of beacons in sight during the IS flight are stochastic because many of the same beacons are deployed inside the space station cabin for good coverage. This problem seems to prevent the conventional perspective-N-point method^13,14^ from directly solving the pose estimation problem of IS. Pattern recognition methods based on features of the images, such as shape context^15,16^ and spectral graph theory^17,18^, are reasonable ways to determine the corresponding relationship between two point sets of different images in most cases. However, these methods seem incapable of solving the problem in the situation of IS navigation. The number of beacons in sight during the IS flight is much smaller than the feature point amount in an image commonly studied; thus, most methods are not robust enough.

Iterative closest point (ICP), which was first introduced in 1992^19^, is an algorithm employed to minimize the difference between two clouds of points. This algorithm is often conducted for point set registration or transformation estimation^20,21^. Although ICP does not directly solve the navigation problem of IS, the core idea of ICP provides an important reference. In the present paper, the imaging model for a multiple field-of-view vision system is established. Based on the imaging model, a novel algorithm called iterative closest imaging point (ICIP), which estimates the attitude and position of the vision system, is proposed. Furthermore, considering the sensitivity of ICP algorithms, a Gaussian mixture model (GMM)-based initialization^22–24^ is utilized. On this basis, a continuous autonomous navigation method using the ICIP algorithm is designed and analyzed by numerical simulation. Experimental results show that ICIP-based navigation operates effectively. This method suits not only the IS navigation inside the space station but also similar application situations, such as indoor, inside tunnels, and underwater.

## ICIP-based Autonomous Navigation Method

### Imaging model

The ideal pinhole model of mapping of a 3D point *P* (Xw, Yw, Zw) to a 2D image *p* (u, v) is given by

1 $${Z}_{c}[\begin{array}{c}u\\ v\\ 1\end{array}]=[\begin{array}{ccc}1/dx & s & {u}_{0}\\ 0 & 1/dy & {v}_{0}\\ 0 & 0 & 1\end{array}]\,[\begin{array}{cccc}f & 0 & 0 & 0\\ 0 & f & 0 & 0\\ 0 & 0 & 1 & 0\end{array}]\,[\begin{array}{cc}{\boldsymbol{R}} & {\boldsymbol{T}}\\ {{\boldsymbol{0}}}^{T} & 1\end{array}]\,[\begin{array}{c}{X}_{W}\\ {Y}_{W}\\ {Z}_{W}\\ 1\end{array}]=[\begin{array}{cccc}{\alpha }_{x} & \gamma  & {u}_{0} & 0\\ 0 & {\alpha }_{y} & {v}_{0} & 0\\ 0 & 0 & 1 & 0\end{array}]\,[\begin{array}{cc}{\boldsymbol{R}} & {\boldsymbol{T}}\\ {{\boldsymbol{0}}}^{T} & 1\end{array}]\,[\begin{array}{c}{X}_{W}\\ {Y}_{W}\\ {Z}_{W}\\ 1\end{array}],$$

where *Z*_*c*_ is the optic axis coordinate of point *P*. *dx* and *dy* are the respective ratio coefficients in the *x* and *y* directions, respectively. *s* and *γ* are the non-orthogonal factors of axes of the image coordinate. (*u*_0_, *v*_0_), *f*, ***R***, and ***T*** are the pixel coordinate of the camera principal point, principal distance of the camera, 3 × 3 rotation matrix, and 3D translation vector, respectively. *α*_*x*_ = *f/dx* and *α*_*y*_ = *f/dy* are the scale factors of the *u*-axis and *v*-axis of the image coordinate, respectively.

On the basis of an ideal pinhole imaging model, the displacement of the principal point, focal length deviation, distortion, and other error factors must be considered in the actual imaging system. In practice, several cameras are attached to form a vision navigation system (VNS) in many cases so that the field of view is extended and the measurement model is optimized. The imaging model is extended based on equation (1) and can be written as

2 $${Z}_{ci}[\begin{array}{c}{u}_{i}\\ {v}_{i}\\ 1\end{array}]=[\begin{array}{cccc}{\alpha }_{xk} & {\gamma }_{k} & {u}_{0k} & 0\\ 0 & {\alpha }_{yk} & {v}_{0k} & 0\\ 0 & 0 & 1 & 0\end{array}]\,[\begin{array}{cc}{{\boldsymbol{C}}}_{b}^{k} & {\boldsymbol{0}}\\ {{\boldsymbol{0}}}^{T} & 1\end{array}]\,[\begin{array}{cc}{{\boldsymbol{C}}}_{w}^{b} & {{\boldsymbol{r}}}_{bw}^{b}-{{\boldsymbol{r}}}_{bk}^{b}\\ {0}^{T} & 1\end{array}]\,[\begin{array}{c}{X}_{Wi}\\ {Y}_{Wi}\\ {Z}_{Wi}\\ 1\end{array}]$$

where *k* = *1*, *2*, *3*, *…* represents the number of cameras; *i* = *1*, *2*, *3*, … represents the number of beacons observed, ${{\boldsymbol{C}}}_{b}^{k}$ is the rotation matrix from the VNS body coordinate to the camera *k* coordinate, ${{\boldsymbol{C}}}_{w}^{b}$ is the rotation matrix from the world coordinate to the VNS body coordinate, and ${{\boldsymbol{r}}}_{bw}^{b}={({t}_{x},{t}_{y},{t}_{z})}^{T}$ and ${{\boldsymbol{r}}}_{bk}^{b}={({r}_{xk},{r}_{yk},{r}_{zk})}^{T}$ are the translation vectors from the origin of the VNS body coordinate to the origins of the world coordinate and camera *k* coordinates, respectively, which are expressed in the VNS body coordinate.

The 6-DOF navigation parameters are the three Euler angles related to ${{\boldsymbol{C}}}_{w}^{b}$ and the three components of ${{\boldsymbol{r}}}_{bw}^{b}$. Let $${{\boldsymbol{A}}}^{k}=[\begin{array}{cccc}{\alpha }_{xk} & {\gamma }_{k} & {u}_{0k} & 0\\ 0 & {\alpha }_{yk} & {v}_{0k} & 0\\ 0 & 0 & 1 & 0\end{array}]\,[\begin{array}{cc}{{\boldsymbol{C}}}_{b}^{k} & {\boldsymbol{0}}\\ {{\boldsymbol{0}}}^{T} & 1\end{array}]={({a}_{ij}^{k})}_{3\times 4}$$ and ${{\boldsymbol{C}}}_{b}^{w}={({r}_{ij}^{k})}_{3\times 3}$, and the following is obtained:

3 $$\{\begin{array}{c}({a}_{11}^{k}-{a}_{31}^{k}{u}_{k})({r}_{11}{X}_{Wi}+{r}_{12}{Y}_{Wi}+{r}_{13}{Z}_{Wi}+{t}_{x}-{r}_{xk})\\ +({a}_{12}^{k}-{a}_{32}^{k}{u}_{k})({r}_{21}{X}_{Wi}+{r}_{22}{Y}_{Wi}+{r}_{23}{Z}_{Wi}+{t}_{y}-{r}_{yk})\\ +({a}_{13}^{k}-{a}_{33}^{k}{u}_{k})({r}_{31}{X}_{Wi}+{r}_{32}{Y}_{Wi}+{r}_{33}{Z}_{Wi}+{t}_{z}-{r}_{zk})=0\\ ({a}_{21}^{k}-{a}_{31}^{k}{v}_{k})({r}_{11}{X}_{Wi}+{r}_{12}{Y}_{Wi}+{r}_{13}{Z}_{Wi}+{t}_{x}-{r}_{xk})\\ +({a}_{22}^{k}-{a}_{32}^{k}{v}_{k})({r}_{21}{X}_{Wi}+{r}_{22}{Y}_{Wi}+{r}_{23}{Z}_{Wi}+{t}_{y}-{r}_{yk})\\ +({a}_{23}^{k}-{a}_{33}^{k}{v}_{k})({r}_{31}{X}_{Wi}+{r}_{32}{Y}_{Wi}+{r}_{33}{Z}_{Wi}+{t}_{z}-{r}_{zk})=0\end{array}$$

Equation (3) is the practical imaging model of the VNS. The pose of the VNS is possibly obtained when many beacons are observed.

### ICIP algorithm statement

Two possible ways exist for a VNS to realize the navigation based on reference beacons. First, when the VNS takes pictures of the environment, it searches for a pose where similar pictures will be observed. This pose is the solution of the navigation parameters. Second, the corresponding relationship between the beacons and the observed images are directly determined through feature matching, and then the pose of the vision system based on the imaging model is calculated. For beacon-based navigation, only several feature points are in the image after feature extraction, causing extreme difficulty in completing pattern matching that utilizes existing methods. Thus, the first method using pose searching is implementable, and the problem becomes proposing a searching strategy.

One of the most widely used point set registration algorithms, ICP has advantages of simple architecture and low computational complexity. The basic principle of ICP is finding the nearest point couples between two point sets, and then minimizing the distance between the point couples by estimating the transformation between them. This process is operated iteratively until locally optimal solutions are found^19^. Later research focuses on the improvement of the origin method through various ways, such as optimizing the point set matching process^25,26^ and improving the optimal estimation algorithm^27^.

Inspired by the core idea of ICP, a novel algorithm called ICIP is proposed. Once the beacon coordinates are known and the vision system parameter is calibrated, the images observed at any pose can be predicted using the imaging model (equation (3)). These predicted images are similar to the obtained real images if the pose is close to the true value. Thus, a roughly corresponding relationship between the beacons and the images is obtained by matching the closest point couples between a predicted image and a real image. In other words, each point couple is assumed as the same image of the beacon. Therefore, the 3D coordinates of beacons and their images are obtained so that an observable measurement equation can be built based on equation (3) once ≥3 beacons are in sight. The optimal solution of the pose can be found using the Levenberg–Marquardt (L-M) method, and then new predicted images are generated at that pose. The process composed of predicting images, matching closest couples, and L-M iteration is repeatedly operated until a locally optimal solution of the pose is found. The distance between two image points *p*_1_(*u*_1_, *v*_1_) and *p*_2_(*u*_2_, *v*_2_) is defined as $d({p}_{1},{p}_{2})=\sqrt{{({u}_{1}-{u}_{2})}^{2}+{({v}_{1}-{v}_{2})}^{2}}$.

The ICIP algorithm is stated as follows:

- The pictures of the current surroundings are taken by the VNS, and the pixel coordinates of the beacon images, namely, data point set ***q***, are recognized through simple feature extraction. The database of the beacons ***P***, namely, the coordinate of each beacon, is given.
- An initial value of the VNS pose is estimated (see Initialization section), and the iteration is initialized by setting *k* = 0.

- *Generating the model point set*: the visible beacon set ${{\boldsymbol{P}}}_{0}^{k}\subseteq {\boldsymbol{P}}$ and the corresponding image point set ${{\boldsymbol{p}}}_{0}^{k}$ at the initial pose $({{\boldsymbol{R}}}_{0}^{k},{{\boldsymbol{t}}}_{0}^{k})$ are predicted based on the beacon position database ***P*** and the imaging model ${{\boldsymbol{p}}}_{0}^{k}={\boldsymbol{g}}({{\boldsymbol{R}}}_{0}^{k},{{\boldsymbol{t}}}_{0}^{k},{{\boldsymbol{P}}}_{0}^{k})$, where ${\boldsymbol{g}}()$ stands for equation (3).
- *Matching the nearest couples*: for each data point ${q}_{i}(u\text{'},v\text{'})\in q$ where *i* = 1, 2, …, and *n* stands for the number of the visible beacon, as well as its image in the picture, find a ${p}_{i}^{k}(u,v)\in {{\boldsymbol{p}}}_{0}^{k}$ such that (s.t.) $d({p}_{i}^{k},{q}_{i})=\,\min$.
- *Estimating the transformation*: the rotation matrix ***R*** ^*k*^ and the translation vector ***t*** ^*k*^, s.t. $\sum _{i=1}^{n}d({\boldsymbol{g}}({{\boldsymbol{R}}}^{k},{{\boldsymbol{t}}}^{k},{P}_{i}^{k}),{q}_{i}^{k})=\,{\rm{\min }}$ are calculated, where ${P}_{i}^{k}({X}_{w},{Y}_{w},{Z}_{w})$ is the 3D coordinate of the corresponding beacon of ${p}_{i}^{k}(u,v)$.
- *Applying the transformation*: ${{\boldsymbol{p}}}^{k}={\boldsymbol{g}}({{\boldsymbol{R}}}^{k},{{\boldsymbol{t}}}^{k},{{\boldsymbol{P}}}^{k})$, where ${{\boldsymbol{P}}}^{k}=\{{P}_{i}^{k},i=1,2,\mathrm{...},n\}$.
- *Examining convergence*: the iteration if ${d}^{k}-{d}^{k+1} < \tau$ is terminated, where $\tau  > 0$ is a present threshold, and

4 $${d}^{k}=\{\begin{array}{c}\frac{1}{n}\sum _{i=1}^{n}d({p}_{i}^{0},{q}_{i}),k=0\\ \frac{1}{n}\sum _{i=1}^{n}d({\boldsymbol{g}}({{\boldsymbol{R}}}^{k},{{\boldsymbol{t}}}^{k},{P}_{i}^{k}),{q}_{i}),k=1,2,\mathrm{...},n\end{array}.$$

**Figure 2:**
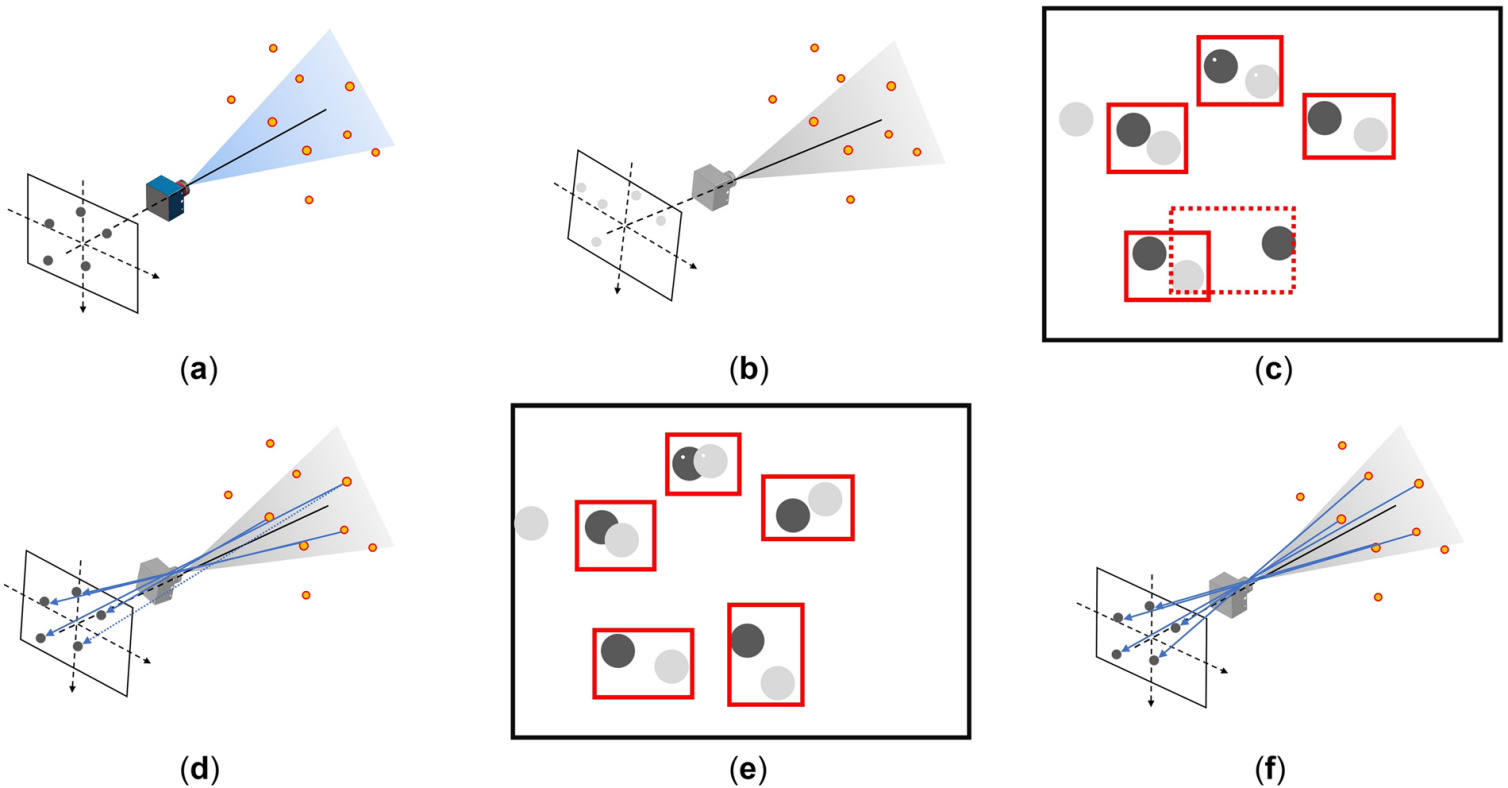
ICIP algorithm process: (**a**)–(**f**) are processed circularly until convergent. (**a**) VNS captures a picture of current surroundings and beacon images are extracted. (**b**) Observed beacon images are predicted based on imaging model at current estimated initial pose. (**c**) Nearest point couples between real beacon images and predicted ones are matched, and a mismatch may occur during this step, which is marked by a dashed frame. (**d**) Measurement equation is formed based on imaging model by assuming that each point couple is the same image of beacon. Optimized solution of the pose is then calculated, and mismatched image is shown by a dashed-line arrow. (**e**) A newly observed beacon image is predicted at the previous optimized pose, and new nearest point couples are matched. As the optimized pose is closer to the true pose, a mismatch may be corrected, and (**f**) a new, more accurate optimized pose is calculated based on previous new point couples.

Otherwise, the transformation in step c is taken as a new initial value: $({{\boldsymbol{R}}}^{k},{{\boldsymbol{t}}}^{k})=({{\boldsymbol{R}}}_{0}^{k+1},{{\boldsymbol{t}}}_{0}^{k+1}),k=k+1$ and the process returns to step a. The process of the algorithm is shown in Fig. 2.

### Convergence theory

*Theorem*: The iterative closest imaging point algorithm always converges monotonically to a local minimum estimated pose.

Proof: For the *k* ^th^ iteration, after ICIP step b, the mean pixel distance error of the closest image point couples *e* ^*k*^ is represented in the following:

5 $${{e}}^{{k}}=\frac{1}{{n}}\sum _{i=1}^{n}{d}({{p}}_{{i}}^{{k}},{{q}}_{{i}}).$$

The optimized mean distance between point couples *d* ^*k*^ after step c is shown in equation (4). The case *d* ^*k*^ ≤ *e* ^*k*^ always occurs because the transformation is estimated to minimize the distance between the closest image point couples.

During the subsequent iteration, a new point set ${p}_{i}^{k+1}(u,v)$ is obtained after step b, forming a new closest corresponding point set of ***q***. Thus, the following is clear: $d({p}_{i}^{k+1},{q}_{i})\le d({\boldsymbol{g}}({{\boldsymbol{R}}}^{k},{{\boldsymbol{t}}}^{k},{P}_{i}^{k}),{q}_{i}),i=1,2,\mathrm{...},n$. According to the definition, the image point distance are positive numbers. Therefore, *e* ^*k+1*^ ≤ *d* ^*k*^, and the following inequality is obtained:

6 $$0\le {d}^{k+1}\le {e}^{k+1}\le {d}^{k}\le {e}^{k}\,{\rm{for}}\,{\rm{all}}\,k.$$

The iteration will converge to the true value as long as the initial estimation is within tolerance.

### ICIP-based navigation method

**Figure 3:**
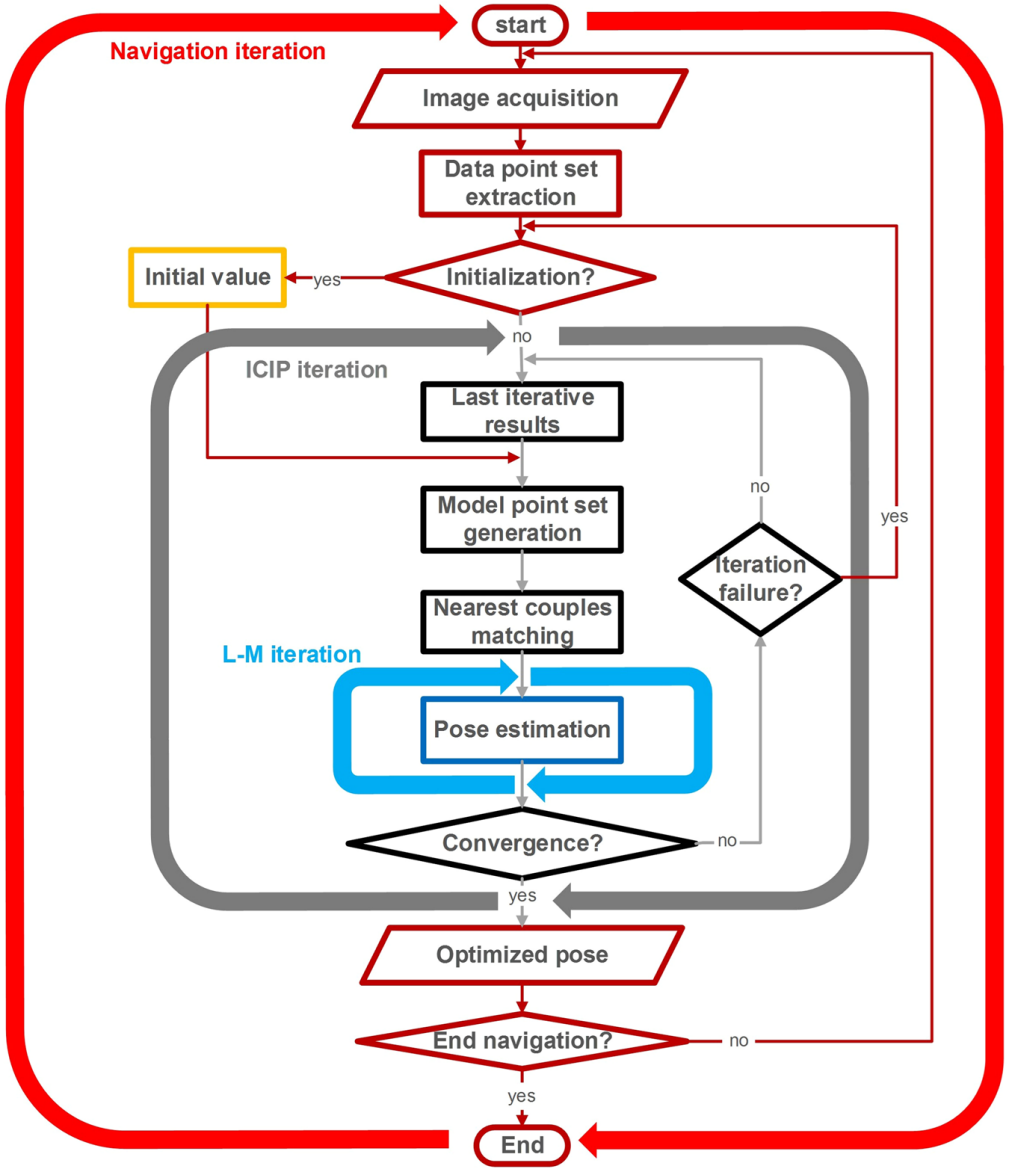
Navigation process based on ICIP algorithm.

A continuous autonomous navigation method utilizing the ICIP algorithm is designed as shown in Fig. 3.

The entire navigation process consists of three levels of iterations. The third level is *L-M iteration* that conducts the pose estimation (marked in blue in Fig. 3). As the estimated pose is not accurate enough, it is taken as the initial value, and another loop containing model point set generation, nearest point couple matching, and transformation estimation is conducted until convergence, which is the second-level iteration: *ICIP iteration* (marked in gray in Fig. 3). The convergent pose estimation is considered the initial value for the next moment, and a new process of image acquisition, data point set extraction, and ICIP iteration is performed. This process is the first-level iteration, namely, *navigation iteration* (marked in red in Fig. 3).

For the first step of the navigation iteration, the initial value is required to estimate an approximate pose. Once the ICIP iteration fails due to accidental factors, the loop breaks and a new one starts from the initialization step. Note that the failure can be determined by the distance threshold and the iteration times. The main influencing factor for convergence of the navigation method is whether the initial value is within tolerance. For the ICIP iteration, if the initial value is not reasonable, falling into a local optimal solution is easy. For the navigation iteration, if the computation speed is slower than the moving speed of the system, the navigation may fail to continue. These problems are discussed in detail in the two following sections.

### Initialization for ICIP

An extended Kalman filter (EKF) framework proposed by Moreno-Noguer *et al*.^22^ is employed to initialize the ICIP. The VNS pose with respect to the beacons are represented as a GMM. Constraining the initial pose of the IS within a certain range is reasonable, and the pose samples can be generated to train the GMM offline. Thereafter, 6-vector Gaussian components $\{{{\bf{x}}}_{1},\mathrm{...},{{\bf{x}}}_{G}\}$, which represents the pose and corresponding covariance matrix $\{{{\boldsymbol{\Sigma }}}_{1}^{x},\mathrm{...},{{\boldsymbol{\Sigma }}}_{G}^{x}\}$ are obtained. The initial pose ***x*** can be obtained by minimizing the following objective function:

7 $$Error({\boldsymbol{x}})=\sum _{({\boldsymbol{x}},{\boldsymbol{P}})\in Matches}\Vert {\boldsymbol{q}}-{\boldsymbol{g}}({\boldsymbol{x}},{\boldsymbol{P}})\Vert +\tau {N}_{nd},$$

where *Matches* is a set of (***P***, ***q***) pairs that represents the correspondence between the 3D beacons ***P*** and their image ***q***. *N*_*nd*_ = |*NotDetected*| denotes beacons for which no match can be established.τ is a penalty term.

The pose search strategy is as follows: for each Gaussian component, the 2D projections ***p*** of the 3D beacons ***P*** are calculated. The potential match is considered in turn such that

8 $${({{\boldsymbol{p}}}_{i}-{{\boldsymbol{q}}}_{i})}^{T}{({{\boldsymbol{\Sigma }}}_{i}^{p})}^{-1}({{\boldsymbol{p}}}_{i}-{{\boldsymbol{q}}}_{i})\le {M}^{2}$$

where ${{\boldsymbol{\Sigma }}}_{i}^{p}={\boldsymbol{J}}({{\boldsymbol{P}}}_{i}){{\boldsymbol{\Sigma }}}_{g}^{x}{({\boldsymbol{J}}({{\boldsymbol{P}}}_{i}))}^{T}$ indicates the error propagation, ${\boldsymbol{J}}({{\boldsymbol{P}}}_{i})$ is the Jacobian matrix of the projection ${\boldsymbol{g}}({\boldsymbol{x}},{{\boldsymbol{P}}}_{i})$, and *M* is a confidence threshold. An EKF framework is then utilized to update the pose estimate and its covariance:

9 $$\begin{array}{c}{{\boldsymbol{x}}}_{g}^{+}={{\boldsymbol{x}}}_{g}+{\boldsymbol{K}}({{\boldsymbol{q}}}_{j}-{\boldsymbol{g}}({{\boldsymbol{x}}}_{g},{{\boldsymbol{P}}}_{i}))\\ {{\boldsymbol{\Sigma }}}_{g}^{x+}=({\boldsymbol{I}}-{\boldsymbol{KJ}}({{\boldsymbol{P}}}_{i})){{\boldsymbol{\Sigma }}}_{g}^{x}\end{array}$$

Only three 3D-to-2D matches are needed to search the pose based on the imaging model. As the three orthogonal cameras form an optimal model, one match from each camera is selected to participate in the update in equation (9). After three match hypotheses, all the 3D beacons in sight are projected onto the image. The *Error* in equation (7) is computed by setting a distance on the image *τ* as the threshold for undetected beacons. The 3D beacons whose projection is farther apart from any 2D features than *τ* are considered as not detected.

This preceding process is repeated over the Gaussian components until a sufficiently small *Error* is obtained. In addition, as shown in Fig. 3, initialization is conducted again while ICIP fails to converge. This process is performed by training a new GMM online based on the recorded previous IS motion parameters. For instance, under non-highly dynamic conditions, the previous pose can be used to estimate a small region where the IS is most likely currently located. This region is regarded as a new, constrained initialization space for the IS. Pose sample within this space is randomly generated to train the GMM and the current pose initial can be found to continue the ICIP iteration.

### Simulation Analysis

To analyze the performance of the proposed navigation method, we conducted numerical simulation using MATLAB. Note that the performance varies under different beacon deployments and movement space conditions. Without loss of generality, the following discussion is based on the same beacon deployment as the IS ground testing system. The VNS is also limited to move inside this system, which is a simulated environment of the space station cabin of 1 × 1 × 1.5 m^3^. The VNS utilizes a configuration of three orthogonal cameras. The system moves inside the given space, takes pictures of the environment, and calculates its own pose utilizing the proposed method, as shown in Fig. 1(c). The simulation conditions are provided in Supplementary Table S1.

#### Initial value sensitivity

The initial value sensitivity of the ICIP algorithm presents two practical significances as previously mentioned. First, at the beginning of the navigation iteration, a reasonable initial estimated pose is required. Thus, the initial value sensitivity submits a request to the accuracy of that estimation. Second, as the optimized solution of the previous navigation iteration is the initial value of the current navigation iteration, the moving and rotational speed of the VNS are limited by the initial value sensitivity. In other words, the displacement of the VNS during the computing time must be within the initial value of allowable range.

**Figure 4:**
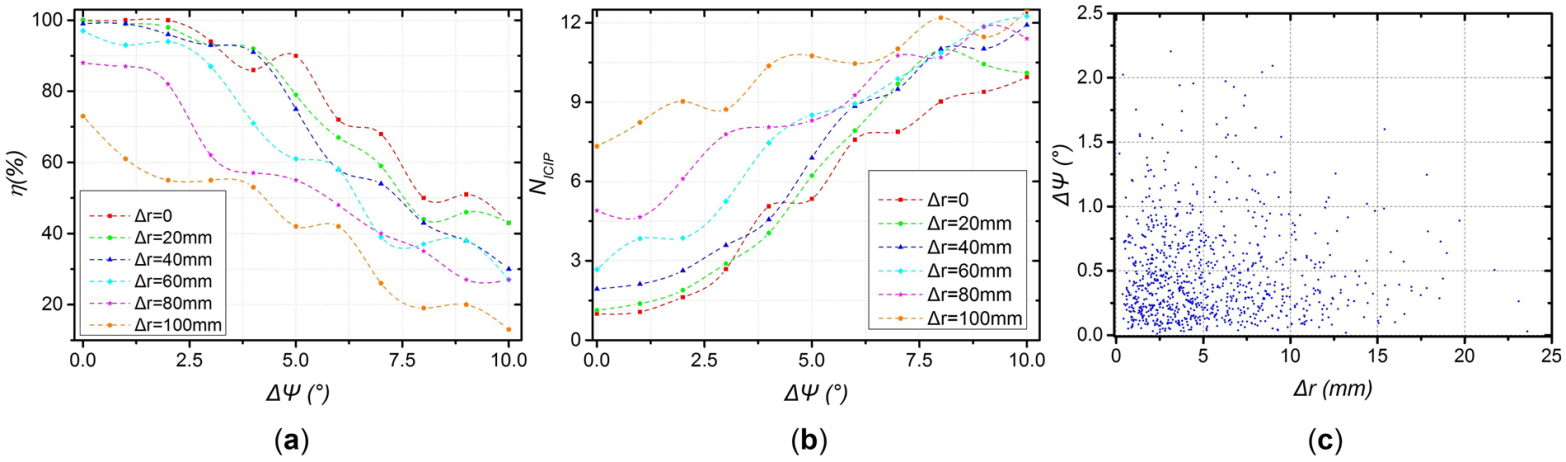
(**a**) Success rate *η* and (**b**) number of ICIP iterations, *N*_*ICIP*_, vs. displacement of VNS. (**c**) Initialization M-C simulation results.

Monte Carlo (M-C) simulation is conducted to obtain statistical results under different initial value error conditions. The “initial value error” refers to the difference in both attitude angle and position between the initial value and the true value, that is, Δ*Ψ* and Δ*r*. As shown in the simulation result in Fig. 4, the success rate *η* decreases and the number of ICIP iterations, *N*_*ICIP*_, increases as Δ*Ψ* and Δ*r* increase. According to Fig. 4(a), a 20-component GMM is trained. The initialization results are shown in Fig. 4(c), revealing that the success rate of the initialization reaches 99.6% in a 10,000-trail M-C simulation. The same conclusion^28^ that the initialization method is robust can be presented based on the simulation. After proper initialization, the pose is refined by ICIP, and the optimized solution is taken as the initial value of the next navigation iteration. Whether the navigation can be continuous mainly depends on the onboard operating speed of the algorithm, which is discussed in the following subsection.

#### Operating speed

A typical process of the navigation iteration after image acquisition is composed of image transmission, image processing and feature extraction, model point set generation, closest point couple matching, and L-M iteration. The operating speed is evaluated by MATLAB R2014a running on a PC (Intel (R) Core (TM) i7-4900 CPU at 3.60 GHz, 8.00 GB RAM).

**Figure 5:**
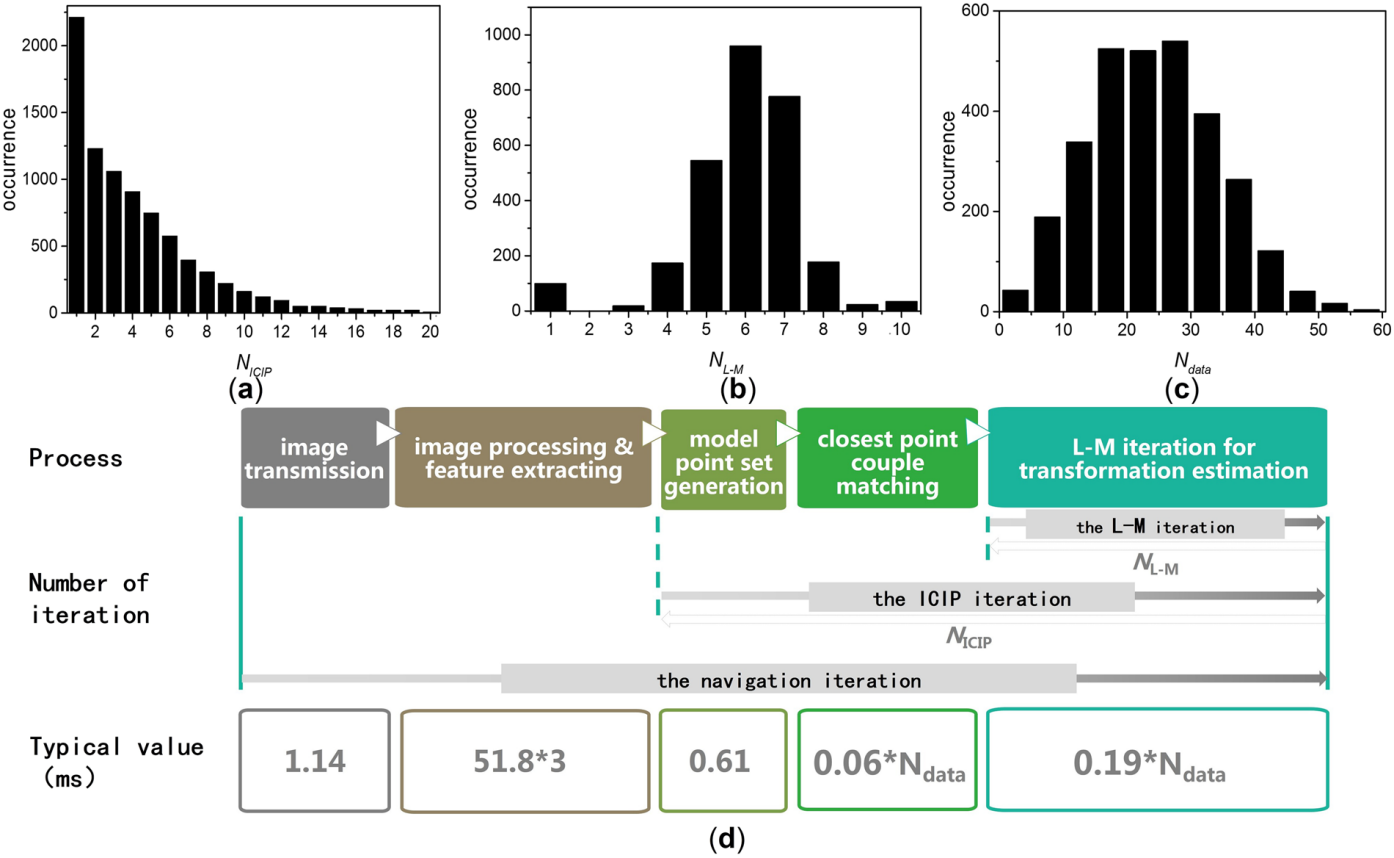
Statistics of (**a**) number of ICIP iteration time *N*_*ICIP*_, (**b**) number of L-M iteration time *N*_*L-M*_, and (**c**) number of in-sight beacons *N*_*data*_. (**d**) is the time taken by each process of ICIP algorithm-based navigation.

As shown in Fig. 5(d), the main time cost lies in the process of image processing and the L-M iteration. For each loop of the navigation iteration, the first two steps in Fig. 5 are performed once, and the last three steps that form the ICIP iteration are performed *N*_*ICIP*_ times depending on the initial value accuracy as shown in Fig. 4(b). The time cost of the closest point couple matching step is proportional to the number of in-sight beacons *N*_*data*_. The duration time of the L-M iteration is proportional to *N*_*data*_ and the number of L-M iteration time *N*_*L-M*_. Under the given beacon deployment, the mean values of *N*_*data*_, *N*_*ICIP*_, and *N*_*L-M*_ are 24.64, 4.06, and 6.01, respectively. The statistical results of these numbers are presented in Fig. 5(a–c). Thus, the time of one navigation iteration loop is approximately 279.25 ms. Under this condition, the navigation system can provide continuous service when the movement is within 7 deg/s and 70 mm/s. The L-M and ICIP iterations consume 40.9% and 43.9% of the total time, respectively.

#### Accuracy

**Figure 6:**
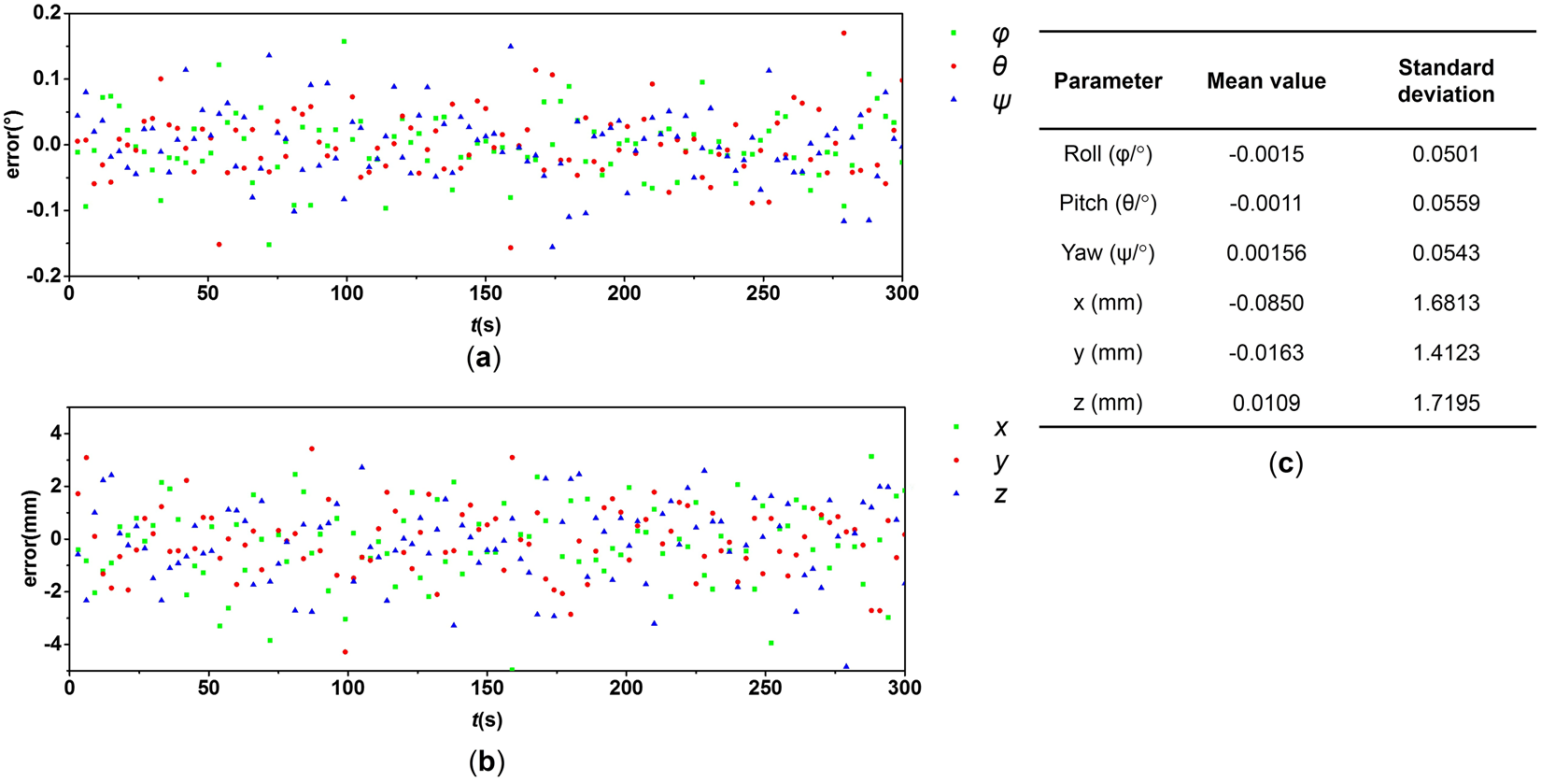
Simulation results of ICIP method: (**a**) attitude error, (**b**) position error, and (**c**) result datasheet.

Accuracy is one of the most important performance indices of a navigation system. In this study, the navigation accuracy is mainly determined by the camera configuration and beacon deployment. Autonomous navigation of IS is simulated while the Δ*Ψ* and Δ*r* are within the allowable range. In the simulation results shown in Fig. 6, the accuracy of the three-axis Euler angles is within 0.06° (1σ) and the three-axis position accuracy is within 1.8 mm (1σ). This result indicates that the proposed method fits the requirement of the IS navigation.

#### Robustness

On the basis of the imaging model equation (2), the 6-DOF pose can be calculated as long as ≥3 beacons are observed. Simulation results show that the navigation method works properly when manually blocking some of the beacons. Thus, the proposed ICIP is robust to occlusion. This property can be considered as an advantage to reduce the computation load by actively choosing only some of the observed beacons to form the imaging model. As shown in Fig. 5(d), the time cost can be reduced when *N*_*data*_ is reduced. However, simulation results also indicate that this improvement in efficiency is at the cost of accuracy because lesser beacons in Eq. 2 lead to larger error propagation. The outliers may cause a convergence problem for the ICP-based algorithm. Therefore, proper design and extraction of beacons is necessary, as previously discussed. Finally, continuous navigation is achieved by obtaining the last estimated pose as the initial value of the current iteration. Thus, once the pose estimation fails, the navigation has to break off. To solve this problem, we design the proposed method to immediately start a new cycle by estimating a new initial value for the current pose and run into the iteration loop (see Fig. 3). As a high initialization success rate can be achieved using the GMM, the navigation can continue in a stable manner. In summary, ICIP algorithm-based navigation is robust to beacon blocking, feature extraction error, and cyclic failure.

### Navigation Experiment

**Figure 7:**
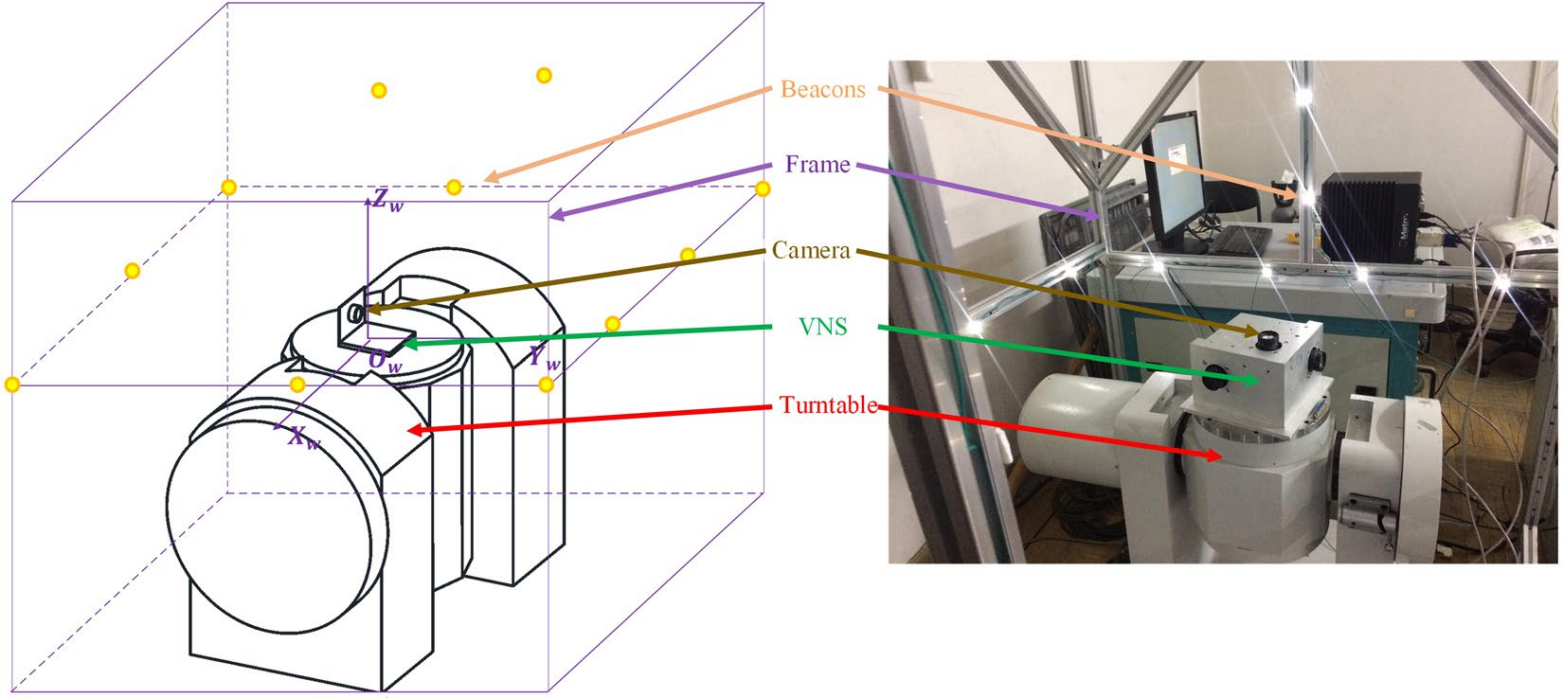
Architecture of navigation experiment.

A demonstration experiment is conducted for further verification. The architecture is shown in Fig. 7.

All coordinates involved in the navigation experiment are right-handed Cartesian coordinates described as follows:

1. The VNS body coordinate *O*_*b*_ *X*_*b*_ *Y*_*b*_ *Z*_*b*_ is fixed on the system frame and defined for easy use.
2. In the turntable coordinate *O*_*t*_ *X*_*t*_ *Y*_*t*_ *Z*_*t*_, *O*_*t*_ is the center of the turntable and *Z*_*t*_ and *X*_*t*_ point to the forward of the turntable main and auxiliary axes, respectively.
3. The world coordinate *O*_*w*_ *X*_*w*_ *Y*_*w*_ *Z*_*w*_ is defined by the beacons and represents the cabin coordinate where the IS operates.
4. The camera coordinates X, Y, and Z are defined based on the imaging model in the *imaging model* section and recorded as *O*_*x*_ *X*_*x*_ *Y*_*x*_ *Z*_*x*_, *O*_*y*_ *X*_*y*_ *Y*_*y*_ *Z*_*y*_, and *O*_*z*_ *X*_*z*_ *Y*_*z*_ *Z*_*z*_, respectively.

LED beacons are fixed on the steel frame surrounding the turntable. The experiment is designed as follows. First, the origin of the world coordinate is defined as the center of the turntable platform, which is the origin of the VNS body coordinate in the original pose. The three axes of the world coordinates *X*_*w*_, *Y*_*w*_, and *Z*_*w*_ are defined parallel to the axes of the turntable coordinates *X*_*t*_, *Y*_*t*_, and *Z*_*t*_, respectively. Subsequently, the position of the beacons is measured using a theodolite. Second, the turntable is controlled to rotate around the *Z*_*t*_ axis, whereas the VNS moves with the turntable, takes pictures of the surrounding environment, and calculates the pose based on the beacons observed. Finally, on the basis of the definition of the coordinates, the position of the turntable platform center does not move during the rotation. The true value of the VNS position is (0, 0, 0) and the true attitude is (0, 0, *ψ*_*t*_), where *ψ*_*t*_ is the angle given by the turntable around its *Z*_*t*_ axis. Therefore, the pose error is obtained as the difference between the calculated pose and the true value.

**Figure 8:**
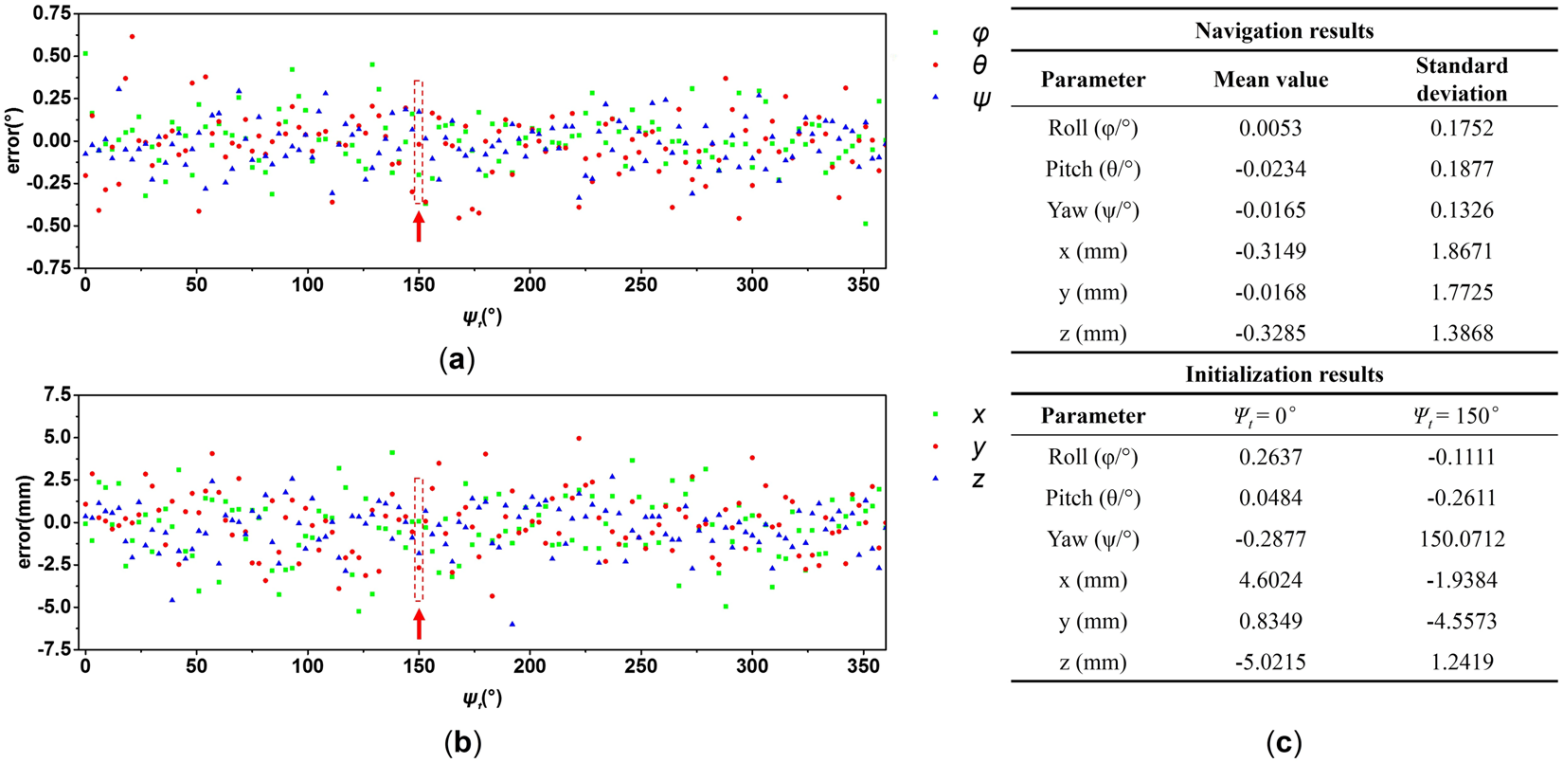
Results of navigation experiment: (**a**) attitude error, (**b**) position error, and (**c**) result datasheet. The red arrow in (**a**) and (**b**) denotes the moment when pose estimation failure is manually added to start and test a new initialization. The corresponding results can be seen in (**c**).

The VNS parameter calibration is conducted using a checkerboard-fixed post-processing calibration, details of which are presented in our previous study^1^. The calibrated parameters are shown in the Supplementary Tables S2 and S3. We use the 902E-1 two-axis testing turntable produced by Beijing Precision Engineering Institute for Aircraft Industry (Beijing, China). The testing turntable is aligned before the experiment, and the accuracy of its angular position is 8′′ in the direction of both axes. The cameras integrated in the VNS are Daheng Image DH-HV1310FM (with resolution of 1280 × 1024, and 18.6 frames under the highest resolution). The theodolite used is Leica TM6100A with an accuracy of 5′′ for both horizontal and vertical angles. Navigation results are shown in Fig. 8.

## Discussion

Experiment results, ways of improvement, and future work are discussed as follows:

1. The experiment results show that the navigation method works properly during the validation. The stand deviations of the three-axis position error are 1.87, 1.77, and 1.39 mm, and the stand deviations of the three-axis attitude error are 0.18, 0.19, and 0.13°, respectively. In this study, only the connective region size and roundness are utilized to justify the reality of the beacon image. Compared with conventional vision navigation based on feature matching, the feature extraction and match process is much simpler, and the required number of beacon images is much smaller than that of the feature points. In particular, pose estimation failure is manually added when the turntable rotates to *ψ*_*t*_ = 150°. Thereafter, another initialization is conducted and the system continues to navigate properly. Therefore, the advantages of low computational complexity, favorable stability, and applicability in an extremely simple environment are verified.
2. The deployment of the navigation beacons significantly influences the accuracy of the system. Thus, future work aims to focus on the beacon deployment strategy and optimization methods. Although theodolite is used because of its high precision, the coordinate error of the beacon cannot be ignored and leads to a measurement error. The concrete operation of deployment and measurement of the beacons will also be the focus. The 3D coordinates of the beacons are essential in this case because the pose with respect to the given frame, that is, the space station cabin frame, is required. As the beacons are measured in the given frame, they establish contacts with the VNS through the imaging process. However, in many applications, only relative positioning is required. The features of the environment need not be measured beforehand but recognized and recorded through machine learning by the VNS. The proposed ICIP algorithm also offers potential application in these cases by using the environment features as the navigation beacons.
3. A promising way to improve the system performance is to apply integrated navigation technology, which we will study in future work. Through a combination of a micro inertial measurement unit (MIMU), the dynamic performance and refresh rate can be improved. Therefore, the movement permissible range of the VNS will be widely extended. The vision navigation results can help control the drift error of the MIMU. Furthermore, the inertial parameters can also help predict the position of the beacon images, thereby hastening the search process and reducing the image process complexity.

## Conclusion

Inspired by the requirement of IS navigation, the ICIP algorithm is proposed in this paper. Based on the imaging model and beacon database, the optimized pose of the VNS can be obtained by the algorithm. The process is similar to adjusting the VNS pose so that it observes images that are most analogous to the real scene. A navigation method is introduced, which utilizes the ICIP algorithm to calculate the current pose and takes it as the initial value of the subsequent iteration. A GMM-based EKF framework is employed to initialize the navigation at the beginning and the failure occurrence moment. Thus, a continuous autonomous navigation is realized. A navigation experiment conducted in the IS ground testing system validates the method. The proposed work fits the navigation inside a cooperative space and has potential for application in unknown environments.

### Data availability statement

The datasets generated during and/or analyzed during the current study are available from the corresponding author on reasonable request.

## Electronic supplementary material

Dataset 1
