# Supplementary material for: A 6-DOF Navigation Method based on Iterative Closest Imaging Point Algorithm
Source: Sci Rep. 2017 Dec 12;7:17414. doi: 10.1038/s41598-017-17768-2 (PMC5727184; doi:10.1038/s41598-017-17768-2)
Supplement: Supplementary file 1 — Dataset 1 [file 41598_2017_17768_MOESM1_ESM.doc]

**A** **6-DOF Navigation Method Based on Iterative Closest Imaging Points Algorithm**

**Shuai Shi**^1,2^**, Zheng You**^1,2,*^**, Kaichun Zhao**^1,2,*^**, Zhaoyao Wang**^1,3^**, Chenguang Ouyang**^1^**, and Yongkui Cao**^1^

^1^Department of Precision Instrument, Tsinghua University, Beijing, China

^2^State Key Laboratory of Precision Measurement Technology and Instruments, Tsinghua University, Beijing, China

^3^China Manned Space Agency, Beijing, China

* [yz-dpi@mail.tsinghua.edu.cn](mailto:yz-dpi@mail.tsinghua.edu.cn); [kaichunz@mail.tsinghua.edu.cn](mailto:kaichunz@mail.tsinghua.edu.cn)

**SUPPLEMENTARY TABLES**

**Supplementary Table S1.** Parameters for simulation on ICIP based navigation method.

| VNS Intrinsic Parameter | | | | | | |
| --- | --- | --- | --- | --- | --- | --- |
| camera |  | *α_x_* (pixel) | *α_y_* (pixel) | *u_0_* (pixel) | *v_0_* (pixel) |  |
| x |  | 1600 | 1600 | 640 | 512 |  |
| y |  | 1600 | 1600 | 640 | 512 |  |
| z |  | 1600 | 1600 | 640 | 512 |  |
| VNS Extrinsic Parameter | | | | | | |
| camera | *φ* (°) | *θ* (°) | *ψ* (°) | *T_x_* (mm) | *T_y_* (mm) | *T_z_* (mm) |
| x | *0* | *90* | *0* | *0* | *0* | *0* |
| y | *-90* | *0* | *0* | *0* | *0* | *0* |
| z | 0 | 0 | 0 | 0 | 0 | 0 |
| VNS measurement error (1σ) | | | | | | |
| 3 *pixel* | | | | | | |
| Total Number of Beacons | | | | | | |
| 229 | | | | | | |

**Supplementary Table S2.** Calibration result of the VNS intrinsic parameters of camera X, Y and Z.

| Parameter | Camera X | | Camera Y | | Camera Z | |
| --- | --- | --- | --- | --- | --- | --- |
|  | Calibration Result | Error | Calibration Result | Error | Calibration Result | Error |
| *α_x_* | 1599.26136 | 1.78966 | 1605.35286 | 1.66249 | 1611.21596 | 2.14183 |
| *α_y_* | 1599.93302 | 1.64101 | 1603.54359 | 1.73608 | 1610.79607 | 2.14219 |
| *u_0_* | 632.61591 | 1.62023 | 619.71227 | 1.80177 | 649.51210 | 1.39920 |
| *v_0_* | 522.17870 | 1.61823 | 505.99361 | 1.93821 | 531.29472 | 1.34749 |
| *α* | 0.00000 | 0.00000 | 0.00000 | 0.00000 | 0.00000 | 0.00000 |
| *kc_1_* | −0.13013 | 0.00336 | −0.11276 | 0.00301 | −0.10481 | 0.00338 |
| *kc_2_* | 0.28701 | 0.02243 | 0.01776 | 0.01776 | 0.15881 | 0.02341 |
| *kc_3_* | −0.00040 | 0.00029 | −0.00031 | 0.00034 | −0.00137 | 0.00021 |
| *kc_4_* | −0.00004 | 0.00031 | 0.00057 | 0.00030 | 0.00184 | 0.00022 |
| *kc_5_* | 0.00000 | 0.00000 | 0.00000 | 0.00000 | 0.00000 | 0.00000 |
| Pixel error | (0.11711, 0.10363) | | (0.11597, 0.12652) | | (0.11593, 0.11232) | |

**Supplementary Table S3.** Calibration result of the VNS extrinsic parameters.

| Rotation Matrix | | | | Position Vector | | | |
| --- | --- | --- | --- | --- | --- | --- | --- |
| Matrix | Euler Angle | Calibration Result (°) | Error (°) | Vector | Component | Calibration Result (mm) | Error (mm) |
| $\mathbf{C}_{t}^{w}$ | *α* | −0.0110 | 0.0485 | $\mathbf{r}_{wb}^{w}$ | $r_{wb,x}^{w}$ | 33.8407 | 0.2932 |
|  | *β* | 0.1263 | 0.0657 |  | $r_{wb,\mathrm{y}}^{w}$ | −128.9401 | 0.2932 |
|  | *γ* | 0.0861 | 0.0443 |  | $r_{wb,\mathrm{z}}^{w}$ | 576.9100 | 0.2456 |
| $\mathbf{C}_{x}^{b}$ | *φ_x_* | −0.5788 | 0.0413 | $\mathbf{r}_{wt}^{w}$ | $r_{wt,x}^{w}$ | 34.0949 | 0.1496 |
|  | *θ_x_* | −90.3523 | 0.0990 |  | $r_{wt,\mathrm{y}}^{w}$ | −128.9281 | 0.1496 |
|  | *ψ_x_* | 0.0395 | 0.0153 |  | $r_{wt,\mathrm{z}}^{w}$ | −684.7134 | 0.1496 |
| $\mathbf{C}_{y}^{b}$ | *φ_y_* | 90.1636 | 0.0301 | $\mathbf{r}_{bx}^{b}$ | $r_{bx,x}^{b}$ | 76.1319 | 0.2529 |
|  | *θ_y_* | −0.5501 | 0.0920 |  | $r_{bx,\mathrm{y}}^{b}$ | −36.8373 | 0.4498 |
|  | *ψ_y_* | 0.1925 | 0.0157 |  | $r_{bx,\mathrm{z}}^{b}$ | 78.8949 | 0.4959 |
| $\mathbf{C}_{z}^{b}$ | *φ_z_* | −0.1076 | 0.0485 | $\mathbf{r}_{by}^{b}$ | $r_{by,x}^{b}$ | −32.5004 | 0.5358 |
|  | *θ_z_* | −0.7211 | 0.0657 |  | $r_{by,\mathrm{y}}^{b}$ | 75.7149 | 0.0825 |
|  | *ψ_z_* | 1.1690 | 0.0443 |  | $r_{by,\mathrm{z}}^{b}$ | 76.9967 | 0.1963 |
|  |  |  |  | $\mathbf{r}_{bz}^{b}$ | $r_{bz,x}^{b}$ | 34.1604 | 0.3097 |
|  |  |  |  |  | $r_{bz,\mathrm{y}}^{b}$ | 30.8251 | 0.3097 |
|  |  |  |  |  | $r_{bz,\mathrm{z}}^{b}$ | 119.7096 | 0.2932 |
